# Using Electronic Health Record Data to Measure the Latent Tuberculosis Infection Care Cascade in Safety-Net Primary Care Clinics

**DOI:** 10.1016/j.focus.2023.100148

**Published:** 2023-09-28

**Authors:** Laura A. Vonnahme, Julia Raykin, Matthew Jones, Jee Oakley, Jon Puro, Adam Langer, Kaylynn Aiona, Robert Belknap, Tracy Ayers, Jonathan Todd, Kathryn Winglee

**Affiliations:** 1 Surveillance, Epidemiology, and Outbreak Investigations Branch, Division of Tuberculosis Elimination, National Center for HIV, Viral Hepatitis, STD, and TB Prevention, Centers for Disease Control and Prevention, Atlanta, Georgia; 2 Peraton, Inc., Reston, Virginia; 3 OCHIN, Portland, Oregon; 4 Public Health Institute at Denver Health, Denver, Colorado; 5 School of Public Health, Oregon Health & Science University, Portland, Oregon

**Keywords:** Tuberculosis infection, primary care, prevention, health data

## Abstract

**Introduction:**

Prevention of tuberculosis disease through diagnosis and treatment of latent tuberculosis infection is critical for achieving tuberculosis elimination in the U.S. Diagnosis and treatment of latent tuberculosis infection in safety-net primary care settings that serve patients at risk for tuberculosis may increase uptake of this prevention effort and accelerate progress toward elimination. Optimizing tuberculosis prevention in these settings requires measuring the latent tuberculosis infection care cascade (testing, diagnosis, and treatment) and identifying gaps to develop solutions to overcome barriers. We used electronic health record data to describe the latent tuberculosis infection care cascade and identify gaps among a network of safety-net primary care clinics.

**Methods:**

Electronic health record data for patients seen in the OCHIN Clinical Network, the largest network of safety-net clinics in the U.S., between 2012 and 2019 were extracted. electronic health record data were used to measure the latent tuberculosis infection care cascade: patients who met tuberculosis screening criteria on the basis of current recommendations were tested for tuberculosis infection, diagnosed with latent tuberculosis infection, and prescribed treatment for latent tuberculosis infection. Outcomes were stratified by diagnostic test and treatment regimen.

**Results:**

Among 1.9 million patients in the analytic cohort, 43.5% met tuberculosis screening criteria, but only 21.4% were tested for latent tuberculosis infection; less than half (40.4%) were tested using an interferon-gamma release assay. Among those with a valid result, 10.5% were diagnosed with latent tuberculosis infection, 29.1% of those were prescribed latent tuberculosis infection treatment, and only 33.6% were prescribed a recommended rifamycin-based regimen.

**Conclusions:**

Electronic health record data can be used to measure the latent tuberculosis infection care cascade. A large proportion of patients in this safety-net clinical network are at high risk for tuberculosis infection. Addressing identified gaps in latent tuberculosis infection testing and treatment may have a direct impact on improving tuberculosis prevention in primary care clinics and accelerate progress toward elimination.

## HIGHLIGHTS

• Latent tuberculosis care cascades can be created using electronic medical record data.
• Care cascades can identify gaps in latent tuberculosis screening, testing, and treatment.
• There is potential to improve tuberculosis screening guideline implementation in primary care.
• Preferred diagnostic test is not used for most tuberculosis infection testing.
• Latent tuberculosis infection treatment prescribing is low, especially preferred regimens.

## INTRODUCTION

Tuberculosis (TB) elimination in the U.S. is defined as a national incidence rate <1 case per 1,000,000 population. Currently, the incidence rate is 27 times the elimination threshold.^1^ More than 80% of TB disease cases in the U.S. are attributed to latent TB infection (LTBI) reactivation despite progression to TB disease being preventable with treatment.^2^^,^^3^ Appropriate screening and treatment of LTBI are critical steps for achieving TB elimination in the U.S. The Centers for Disease Control and Prevention (CDC) and the U.S. Preventive Services Task Force (USPSTF) recommend TB screening for populations at increased risk for infection, progression to TB disease, or transmission to others. These include people who were born or lived outside the U.S. in countries where TB is more common, individuals who are immunocompromised such as persons living with HIV, and those who live or have lived in high-risk congregate settings such as homeless shelters, prisons, or jails.^4^, ^5^, ^6^

Primary care clinics are important settings for LTBI screening and treatment. Safety-net clinics deliver primary healthcare services to patients who experience barriers to accessing care due to insufficient insurance coverage, geographic isolation, language and culture, mental illness, or homelessness.^7^^,^^8^ Most safety-net clinics are community health centers, which serve more than 30 million persons in the U.S.; 65% of these persons belong to racial or ethnic minority groups, 1.3 million persons experience homelessness, and 7 million persons are best served in a language other than English.^9^^,^^10^ Safety-net primary care clinics are especially relevant for LTBI because they serve populations at higher risk for TB infection, such as non-U.S.–born persons and persons experiencing homelessness.^9^, ^10^, ^11^, ^12^, ^13^, ^14^, ^15^, ^16^, ^17^, ^18^, ^19^ They are key settings to engage in efforts to expand TB screening, testing, and treatment.

Electronic health record (EHR) data have the potential to identify persons who should be tested for LTBI and their outcomes through the cascade of care in safety-net clinics. OCHIN is a nonprofit health information technology collaborative that provides linked EHR services to safety-net clinics across 30 states, making it the largest network of safety-net clinics in the U.S.^20^ OCHIN maintains an EHR research database of primary care delivery data from safety-net clinics and includes information from more than 6 million patient records that are demographically similar to the national profile of patients in community health centers.^9^^,^^21^ OCHIN utilizes a common data model to combine EHR data from clinics into this research-ready database that contains aggregated, longitudinal data starting in 2004.^22^

The LTBI care cascade (LCC) is a tool that measures the progression of patients across the care continuum from identifying people at risk for TB infection through treatment completion, with several steps in between.^23^ The LCC is a useful tool to improve TB prevention and identify steps where patients are lost, which can be used to improve TB prevention in clinics and healthcare systems.^23^, ^24^, ^25^ However, measuring the LCC can be difficult and has not been well characterized outside of public health clinics or specific populations (e.g., migrants, persons experiencing homelessness, contacts to TB cases), nor have EHR data been used previously to characterize a complete LCC.^23^^,^^26^ We used a cohort from the OCHIN EHR research data warehouse to characterize the LCC among their network of safety-net clinics and to identify opportunities for potential future interventions to improve TB infection screening, diagnosis, and treatment.

## METHODS

### Study Sample

EHR data were extracted for patients with any encounter at an OCHIN-member clinic between January 1, 2012 and December 31, 2019. The analytic cohort was limited to patients considered established in primary care, defined as at least 2 ambulatory visits (in-person clinic visits) to the same OCHIN-member clinic before or during the study period. Data extracted included demographics, medical risk factors, laboratory results related to LTBI diagnostic tests, diagnostic codes (ICD-9 and ICD-10) associated with the patient's visit history or active problem list, and prescription drug records. Data elements contained in the data set were not restricted on the basis of encounter date, and all patient history up until September 2020 (when data were extracted) was included in the data cohort; patient history from before 2012 was limited and was not considered inclusive of all patient history.

### Measures

The first step in the LCC was to identify patients who should be tested for TB infection. We applied screening criteria adapted from the California Adult Tuberculosis Risk Assessment,^27^ a simplified risk assessment tool based on CDC and USPSTF TB screening guidelines^4^, ^5^, ^6^ that recommends screening for patients who meet 1 or more of the following criteria: (1) birth, travel, or residence in any country other than the U.S., Canada, Australia, New Zealand, or a country in western or northern Europe for at least 1 month; (2) immunosuppression—current or planned—due to HIV infection, organ transplantation, or treatment with TNF-α antagonists, steroids, or other immunosuppressive medication; or (3) known close contact to someone with infectious TB disease during their lifetime. To align with the USPSTF recommendations,^4^ we also included individuals who were likely staying in congregate settings.

EHR variables were not always available to match exact risk criteria. Table 1 describes the sources referenced, screening criteria, and equivalent definitions and variables in the EHR. For the first criterion, country of birth was the preferred variable as a surrogate to identify people who had lived outside the U.S. owing to birth, travel, or residence; among patients without country of birth records, non-English language preference was used as a proxy. Preferred language has been found to be a reasonable proxy for non-U.S.–born persons who meet screening criteria for LTBI.^28^^,^^29^ Because we relied on preferred language, we generalized all non-U.S.–born patients with a recorded country of birth to be at risk.

**Table 1:** Tuberculosis Screening Recommendation Criteria Definitions Used in the OCHIN Electronic Health Record Data Cohort

Source	Screening criterion	Screening criteria equivalent in EHR	EHR variable(s) used
**California Adult Tuberculosis Risk Assessment**^a^	Birth, travel, or residence in any country other than the U.S., Canada, Australia, New Zealand, or a country in western or northern Europe for at least 1 month	Birth in any country other than the U.S. or If country of birth missing, non-English language preference	Country of birth and preferred language
	Immunosuppression—current or planned—due to HIV infection, organ transplantation, or treatment with TNF-α antagonists, steroids or other immunosuppressive medications	HIV	HIV ICD codes: 042, 079.53, 795.71, and V08 (ICD-9) or B20, B97.35, Z21, and 098.7x (ICD-10)^b^
		Organ transplantation	Organ transplant ICD codes: V42.x (ICD-9) or Z94 (ICD-10)^b^
		Aftercare after organ transplantation	Aftercare after organ transplantation ICD codes: V58.44x (ICD-9) or Z48.298 (ICD-10)^b^
		Individuals prescribed immunosuppressive medications	Immunosuppressive drugs prescribed ever before the end of the study period^c^
	Known close contact to an infectious TB case during lifetime	Close contact to a person with infectious TB disease	TB close contact ICD codes: V01.1 (ICD-9), Z20.1 (ICD-10)^b^
**U.S. Preventive Services Task Force recommendations**	Persons who live in or have lived in high-risk congregate settings (e.g., homeless shelters and correctional facilities)	Homelessness status	Ever homeless on or before the end of the study period as indicated by registration variable or in social history. Homelessness ICD codes: V60.0 and V60.1 (ICD-9) or Z59.0x (ICD-10)^b^
		Encounter in a correctional facility	Ever had an encounter on or before the end of the study period in a facility in the OCHIN collaborative that is listed as correctional

a This assessment follows U.S. Preventive Services Task Force and Centers for Disease Control and Prevention recommendations.^4^, ^5^, ^6^

b Diagnostic codes present in visit history and/or active at any time on patient problem list entries ever before the end of the study period.

c Abatacept, adalimumab, anakinra, baricitinib, brodamulab, canakinumab, certolizumab pegol, etanercept, golimumab, guselkumab, infliximab, ixekinumab, methylprednisolone, prednisolone, prednisone (nontopical), risankizumab, rituximab, sariliumab, secukinumab, tildrakizumab, tocilizumab, tofacitinib, upadicitinib, and ustekinumab; drug and brand names were searched for in patient prescription records. EHR, electronic health record.

For the second criterion, *immunosuppression* was defined as patients who ever had or currently have an HIV diagnosis, currently have an organ transplant, or currently are undergoing aftercare after organ transplantation on the basis of the presence of specific ICD-9 and ICD-10 diagnostic codes recorded in patients’ EHR. Also included were individuals ever prescribed immunosuppressive medications (Table 1, footnote c) that place individuals at higher risk for TB reactivation.^30^^,^^31^ Both generic and brand name drugs were searched for in patient prescription drug records. Finally, close contacts to someone with TB disease were identified by a specific diagnostic code recorded in the patients’ EHR.

We also included persons who live in or have lived in high-risk congregate settings by including individuals who reported ever experiencing homelessness and individuals who ever had an encounter at an OCHIN-member clinic that is associated with a correctional facility. Homelessness status was determined by a registration variable and social history variable in the EHR; these variables are updated on the basis of clinic-specific protocols. We also searched for homelessness ICD codes recorded in patients’ EHRs. When a criterion was defined by the presence of an ICD code or a drug prescription, patients were considered to have met specific criteria if the ICD code or drug prescribed was present in their record at any point up to the end of the study period.

After determining the proportion of the cohort who met screening criteria, we removed patients from the LCC who had an ICD diagnostic code for (1) TB disease or (2) a previous history of TB because they would not have been eligible for LTBI testing or treatment. Patients with these codes were removed regardless of whether they also had an ICD diagnostic code for LTBI. Next in the LCC, we determined whether patients had received a TB infection diagnostic test ever until the end of the study period: an interferon-gamma release assay (IGRA) test, including any generation of QuantiFERON TB test or T-SPOT TB test or a tuberculin skin test (TST). Among those tested, we determined the proportion who had a valid diagnostic result, defined as a positive or negative result. We defined a positive result as at least 1 positive IGRA test result (if multiple were performed) or at least 1 positive TST when only TST results were available. A detailed diagram describing how diagnostic test results were defined is available in Appendix Figure 1 (available online).

We defined patients diagnosed with LTBI in the LCC as those with an LTBI diagnostic code in their visit diagnosis history or active at any time on the problem list after a positive diagnostic test in their EHR. A positive diagnostic test is not sufficient for diagnosing LTBI; a diagnosis requires excluding TB disease through a symptom review, a focused physical examination, and a chest radiograph.^32^ However, EHR variables that would allow identification of patient symptom reviews as having occurred or results of a chest radiograph, without requiring individual chart review, do not exist.

Among those diagnosed with LTBI, we determined the proportion who were prescribed an LTBI treatment regimen. We identified recommended LTBI treatment regimens including isoniazid and rifapentine, isoniazid alone, rifampin or rifabutin alone, or isoniazid and rifampin or rifabutin.^33^, ^34^, ^35^ For a drug to be considered part of an LTBI treatment regimen, it had to be prescribed after a positive TB diagnostic test. To reduce potential misclassification of TB disease treatment regimens as LTBI regimens, the regimens mentioned earlier had to be prescribed without the following drugs: pyrazinamide, ethambutol, cycloserine, ethionamide, clofazimine, or linezolid. For regimens where multiple drugs were prescribed, drugs had to be prescribed on the same day. If a patient was prescribed more than 1 LTBI regimen, we deferred to the most recently prescribed regimen. Because rifampin and rifabutin can be used to treat other conditions,^36^^,^^37^ an algorithm was created to determine whether these prescriptions were part of a treatment regimen for TB disease or LTBI and is available in Appendix Methods and Appendix Tables 1 and 2 (available online). Treatment initiation and completion could not be assessed using standard EHR data elements captured.

This activity was reviewed by CDC and was conducted consistent with applicable federal law and CDC policy (e.g., 45 C.F.R. part 46.102[l][2], 21 C.F.R. part 56; 42 U.S.C. §241[d]; 5 U.S.C. §552a; 44 U.S.C. §3,501 et seq). The activity was determined to not be human subjects research because the primary intent of the analysis was routine disease surveillance, and data were used only for disease control programs or policy purposes.

## RESULTS

A total of 1,973,017 patients were included in the analytic cohort owing to having had at least 2 ambulatory visits to the same OCHIN primary care facility before or during the study. Most of the analytic cohort were aged 25–44 years (57.9%), and 56.2% (*n*=1,109,252) were female. A total of 32% identified as Hispanic; 41.3% were non-Hispanic White, 15.7% were non-Hispanic Black, 4.8% were Asian; <1% were Native Hawaiian or other Pacific Islander or American Indian or Alaskan Native. Nativity or country of birth was frequently missing (87.3%). Preferred language was captured for 98.2% of the analytic cohort, and 26.8% preferred a non-English language. The demographics for the analytic cohort are described in Table 2.

**Table 2:** Characteristics of Patients With 2 or More Ambulatory Visits to Same OCHIN Primary Care Clinic, 2012–2019

Patient characteristic	Total analytic cohort *N*=1,973,017		Met screening criteria^a^*n*=858,767		LTBI diagnosis^b^*n*=16,465		Prescribed LTBI treatment^c^*n*=4,791	
	*n*	%	*n*	%	*n*	%	*n*	%
Age (years)^d^								
0–4	83,412	4.2	30,342	3.5	94	0.6	37	0.8
5–14	239,937	12.2	97,298	11.3	798	4.8	291	6.1
15–24	267,341	13.5	91,027	10.6	1,564	9.5	479	10.0
25–44	605,815	30.7	257,577	30.0	6,198	37.6	1,782	37.2
45–64	536,536	27.2	274,239	31.9	5,586	33.9	1,597	33.3
≥65	239,974	12.2	108,284	12.6	2,225	13.5	605	12.6
Unknown/missing	2	0.0	0	0.0	0	0.0	0	0.0
Sex								
Male	863,493	43.8	385,017	44.8	7,113	43.2	2,103	43.9
Female	1,109,252	56.2	473,654	55.2	9,351	56.8	2,688	56.1
Other	192	0.0	68	0.0	1	0.0	0	0.0
Unknown	80	0.0	28	0.0	0	0.0	0	0.0
Race/ethnicity^e^								
Hispanic	631,726	32.0	428,042	49.8	7,430	45.1	2,653	55.4
White, not of Hispanic origin	815,365	41.3	225,665	26.3	1,904	11.6	408	8.5
Black, not of Hispanic origin	310,337	15.7	110,227	12.8	3,402	20.7	818	17.1
Asian	94,655	4.8	59,989	7.0	3,157	19.2	762	15.9
Native Hawaiian or other Pacific Islander	8,332	0.4	2,771	0.3	66	0.4	20	0.4
American Indian or Alaskan Native	10,958	0.6	4,025	0.5	27	0.2	9	0.2
Multiple race	16,148	0.8	4,639	0.5	40	0.2	7	0.1
Unknown/missing race	85,496	4.3	23,409	2.7	439	2.7	114	2.4
Positive TB diagnostic test	26,087	1.3	21,448	2.5	16,465	100.0	4,791	100.0
TST^f^	11,348	43.5	8,830	41.2	6,120	37.2	1,181	24.7
Interferon-gamma release assay (IGRA)^f^	14,739	56.5	12,618	58.8	10,345	62.8	3,610	75.3
Diabetes^g^^,^^h^	236,635	12.0	128,909	15.0	2,778	16.9	809	16.9
Screening recommendation criterion								
Nativity^i^								
U.S. born	132,484	6.7	31,566	3.7	390	2.4	157	3.3
Non-U.S. born	117,468	6.0	117,468	13.7	6,146	37.3	2,267	47.3
Unknown	1,723,065	87.3	709,733	82.6	9,929	60.3	2,367	49.4
Language preference								
English	1,409,652	71.4	339,411	39.5	3,175	19.3	975	20.4
Non-English	528,022	26.8	507,519	59.1	13,209	80.2	3,804	79.4
Unknown	35,343	1.8	11,837	1.4	81	0.5	12	0.3
Immunosuppression								
HIV^h^^,^^j^	22,788	1.2	22,788	2.7	637	3.9	254	5.3
Prescribed immunosuppressive medication^k^	199,578	10.1	199,578	23.2	1,653	10.0	511	10.7
Organ transplantation/aftercare after organ transplantation^h^^,^^l^	2,900	0.1	2,900	0.3	17	0.1	4	0.1
Close contact with a person with infectious TB disease^h^^,^^m^	5,536	0.3	5,536	0.6	521	3.2	210	4.4
Ever experienced homelessness^h^^,^^n^	191,693	9.7	191,693	22.3	2,801	17.0	821	17.1
Ever had an encounter in a correctional facility^o^	34,221	1.7	34,221	4.0	336	2.0	48	1.0

a Patients who met screening criteria described in Table 1.

b Patients with an LTBI diagnosis as described in the LTBI care cascade in Figure 1.

c Patients who were offered LTBI treatment as described in the LTBI care cascade in Figure 1.

d As of January 1, 2020.

e Persons who identified as Hispanic were categorized as Hispanic regardless of race. Persons who did not identify as Hispanic or had an unknown ethnicity were categorized by race.

f Denominator is total positive TB diagnostic tests.

g Diabetes ICD codes: 249.0x, 250.0x (ICD-9), E08.x, E09.x, E10.x, E11.x, E13.x (ICD-10).

h Diagnostic codes present in visit history and/or active at any time on patient problem list entries ever before the end of the study period.

i Based on recorded country of birth.

j HIV ICD codes: 042, 079.53, 795.71, and V08 (ICD-9) or B20, B97.35, Z21, and 098.7x (ICD-10).

k Prescribed one of the following ever before the end of the study period: abatacept, adalimumab, anakinra, baricitinib, brodamulab, canakinumab, certolizumab pegol, etanercept, golimumab, guselkumab, infliximab, ixekinumab, methylprednisolone, prednisolone, prednisone (nontopical), risankizumab, rituximab, sariliumab, secukinumab, tildrakizumab, tocilizumab, tofacitinib, upadicitinib, and ustekinumab; drug and brand names were searched for in patient prescription records.

l Organ transplant ICD codes: V42.x (ICD-9) or Z94.x (ICD-10); aftercare after organ transplant codes: V58.44x (ICD-9) or Z48.2xx (ICD-10).

m TB close contact ICD codes: V01.1 (ICD-9) and Z20.1 (ICD-10).

n Patient ever homeless on or before the end of the study period as indicated by registration variable or in social history or homelessness ICD code (V60.0 and V60.1 [ICD-9] or Z59.0x [ICD-10]) present in visit history or active at any time on patient problem list entries ever before the end of the study period.

o Patient ever had an encounter on or before the end of the study period in a facility in the OCHIN collaborative that is listed as correctional. LTBI, latent tuberculosis infection; TB, tuberculosis; TST, tuberculin skin test.

Among the analytic cohort, 43.5% (*n*=858,767) met screening criteria (Figure 1). A total of 13.7% (*n*=117,468) reported a non-U.S. country of birth, 59.1% (507,519) reported a non-English language preference; 2.7% (*n*=22,788) had an HIV diagnosis, 23.2% (*n*=199,578) were prescribed an immunosuppressive medication, 22.3% (*n*=191,693) had experienced homelessness, and 4.0% (*n*=34,221) had a clinical encounter in a correctional facility (Table 2). Among those who met our screening criteria, 0.8% (*n*=7,198) were removed from the LCC because of an ICD code for either TB disease (*n*=6,083) or a personal history of TB disease in their medical record (*n*=1,610); 495 patients had an ICD code for both.

**Figure 1:**
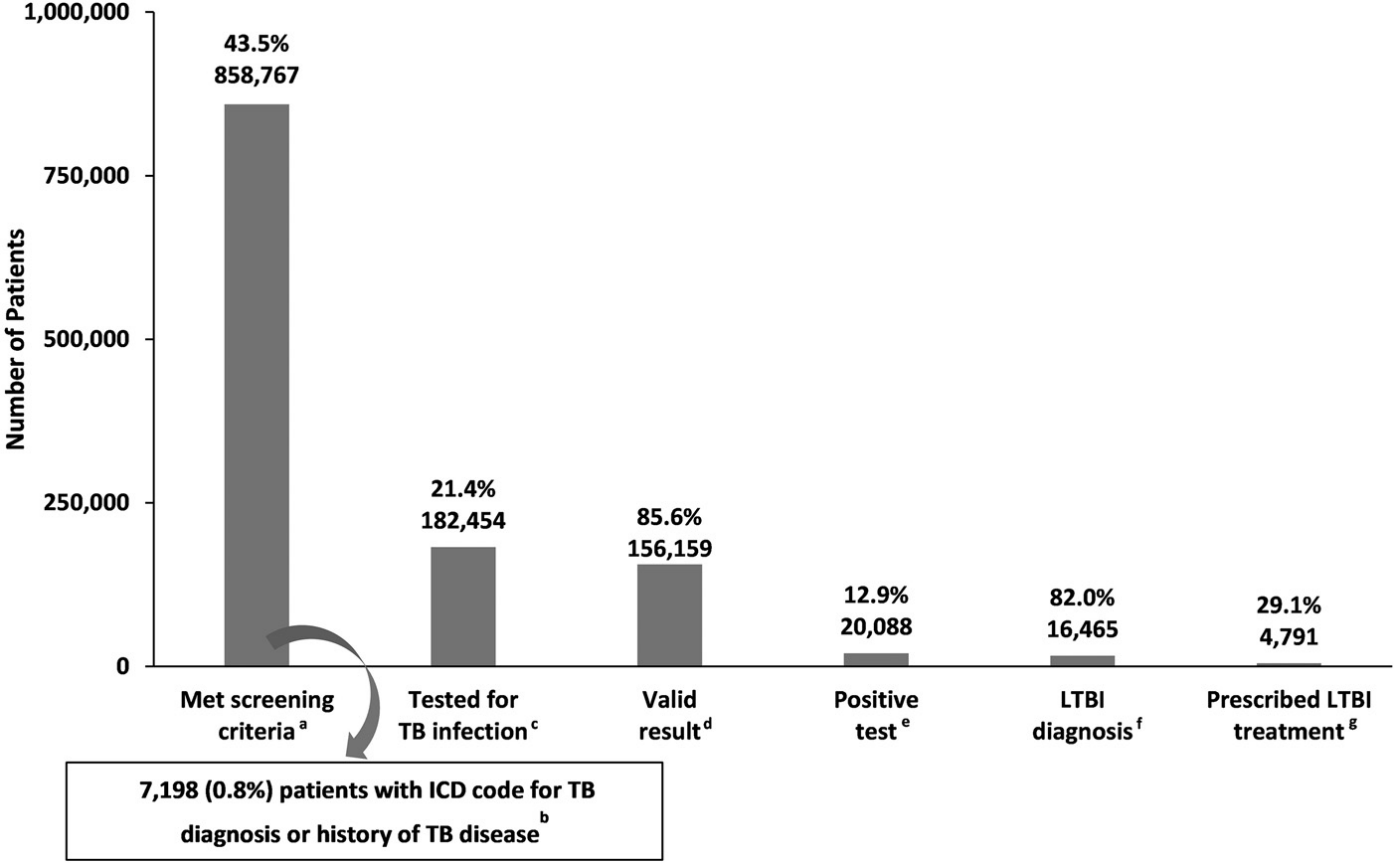
LTBI care cascade among patients with 2 or more ambulatory visits to the same OCHIN primary care clinic, 2012–2019. ^a^Patients who met screening criteria described in Table 1. ^b^TB disease diagnostic codes, including 010, 011, 012, 013, 014, 015, 016, 017, and 018 (ICD-9) or A15, A16, A17, A18, and A19 (ICD-10). Personal history of TB codes includes V12.01 (ICD-9) or Z86.11 (ICD-10). ^c^IGRA test or a TST. ^d^*Valid result* defined as positive or negative. ^e^If patient had any IGRA result that was valid (positive or negative), the result of that test was used to determine the patient's test result. Regardless of multiple IGRA tests and results, if a patient had any positive IGRA test, they were considered LTBI test positive. If a patient had no valid IGRA result, then a patient with any positive TST was considered to have tested positive. ^f^LTBI diagnostic codes, including 795.51 and 795.52 (ICD-9) or R76.11 and R76.12, Z22.7 (ICD-10). ^g^Isoniazid and rifapentine, isoniazid alone, rifampin or rifabutin alone, or isoniazid and rifampin or rifabutin, where rifampin and rifabutin dosages are consistent with a TB disease or LTBI treatment regimen. IGRA, interferon-gamma release assay; LTBI, latent tuberculosis infection; TB, tuberculosis; TST, tuberculin skin test.

Among those patients who met screening criteria and were eligible to continue through the LCC, 21.4% (*n*=182,454) were tested for TB infection. Over half of the total tests performed were TSTs (59.6%, *n*=115,867), with 40.4% (*n*=78,665) of testing being performed by IGRA. Among those tested, 85.6% (*n*=156,159) had a valid test result. Approximately one fourth (23.8%) of TSTs performed did not have a result recorded that could be readily identified; 96.6% (*n*=75,989) of IGRAs performed had valid results. Among those with a valid diagnostic result, 12.9% (*n*=20,088) had a positive result, and 82.0% (*n*=16,465) of those also had an ICD code for LTBI. Thus, 10.5% (*n*=16,465) of patients who met screening criteria and had a valid test result met our LTBI diagnosis criteria. Country of birth was unknown for most of those diagnosed with LTBI (60.3%, *n*=9,929); 80.8% (*n*=13,009) reported a non-English language preference.

Among those diagnosed with LTBI, 29.1% (*n*=4,791) were prescribed an LTBI regimen. The majority (66.4%, *n*=3,180) were prescribed isoniazid alone, 25.3% (*n*=1,214) were prescribed rifampin or rifabutin alone, 7.4% (*n*=356) were prescribed isoniazid and rifapentine, and 0.9% (*n*=41) were prescribed isoniazid and rifampin or rifabutin. Among those prescribed an LTBI regimen, approximately half (50.6%) had a country of birth recorded, with 95% being non-U.S. born; 80% reported non-English language preference.

## DISCUSSION

We created a novel methodology for constructing an LCC using EHR data. Previous studies have used EHR data to determine the proportion who met TB screening criteria, were tested, were diagnosed with LTBI, or were prescribed treatment, but none have reported on the LCC in totality.^28^^,^^38^, ^39^, ^40^ In addition, previous screening criteria definitions were limited by the study population or setting or based solely on preferred language and self-reported nationality.^23^^,^^26^^,^^28^ Our definition was applied to a broad cross-sectional cohort that also included individuals who met criteria owing to immunosuppression, close contact to a TB case, or history of homelessness or incarceration. This is the first study to characterize the LCC across a large network of primary care safety-net clinics; thus, setting a baseline for the at-risk population seeking care in this setting and how TB screening and diagnostic recommendations are being implemented.

On the basis of CDC and USPSTF screening criteria, a large proportion of this clinical network patient population is at high risk for TB infection because 44% of the cohort met the recommended TB screening criteria on the basis of available EHR data. The OCHIN cohort was reflective of a high-risk population; 10% of patients with a valid test result being diagnosed with LTBI is slightly lower than national estimates of TB infection prevalence among non-U.S.–born persons and higher than the estimate of 5% among the U.S. general population.^41^ Another indicator that this population is at high risk for TB infection is that approximately 0.7% of those who met screening criteria were diagnosed with TB disease at some point during the study period. Assuming the population that met screening criteria remained stable throughout this period, that represents a TB incidence rate of 88.5 of 100,000 annually among those who met screening criteria, higher than the reported incidence rate of 34.0/100,000 among non-U.S.–born individuals reported in 2019. Thus, the OCHIN Clinical Network represents an appropriate study population for understanding LTBI testing and diagnosis in patients at high risk of TB infection and for potential interventions to increase screening, improve diagnostic testing, and increase treatment for LTBI.

Our findings demonstrate that there is potential for improvement in TB screening, diagnostic testing, and treatment in this network of clinics. More than 78% of patients who met our screening criteria were not tested for infection. Most testing was performed by TST, which presents an opportunity for increased use of the IGRA diagnostic test, the preferred test in a population who met screening criteria based on non-U.S. birth and other risk factors such as experiencing homelessness.^42^^,^^43^ Treatment prescription was also low in the cohort, and only 33% were prescribed a rifamycin-based treatment regimen. This may reflect that U.S. guidelines for preference of shorter, rifamycin-based therapy to isoniazid monotherapy were not published until 2020.^33^ However, in 2011, CDC recommended once-weekly isoniazid and rifapentine for 12 weeks (3HP) by directly observed therapy as an equal alternative to other regimens;^35^ the recommendations were further expanded in 2018 to include additional populations and types of therapy administration.^34^ Thus, the large proportion of OCHIN patients being prescribed longer-course treatment with isoniazid monotherapy may also represent an implementation gap to be further explored between CDC recommendations and a change in clinical care to using shorter-course therapy.

### Limitations

There were limitations to our study. Although this was a large sample, results are not generalizable to all safety-net clinics in the U.S., and the population is not representative of the overall U.S. population at risk for TB infection. In addition, it is possible that some patients did not go through steps in the LCC successively, for example, a diagnostic ICD code for LTBI might have been entered into a patient EHR in the absence of a positive test, causing us to underestimate some steps. Another limitation is that our evaluation of the LCC was limited to routine EHR data that were captured in structured fields. We also could not determine whether there were specific reasons that steps were not completed. For example, we could not determine whether testing or treatment was offered and declined or whether the treatment that was prescribed was initiated or completed by the patient. In addition, how and what data are routinely captured in an EHR can vary across healthcare settings and across providers within a practice.

Our approach for determining patients’ risk for infection was an innovative methodology but one that likely underestimated the proportion in the cohort who met screening criteria. Country of birth was missing for most patients, and EHR data do not capture information on travel or prior residence outside the U.S. In addition, although non-English language preference is a reasonable proxy for non-U.S.–born persons who may be at increased risk, many non-U.S.–born persons are proficient English speakers and may not have an alternate preferred language in the EHR.^28^^,^^29^^,^^44^^,^^45^ Our methodology was also not able to capture a complete social history of homelessness or incarceration. Finally, we may have underestimated the number of people diagnosed with LTBI by requiring it to be listed as an ICD code.

## CONCLUSIONS

Our results show that an LCC can be constructed using available EHR data from a large network of primary care safety-net clinics. We identified opportunities for improvements in EHR data, such as routinely capturing country of birth as a surrogate measure of risk for TB exposure. We also identified several opportunities in the OCHIN Clinical Network for improvements, including increased testing among patients with potential risk and increased use of the current, recommended diagnostic tests and treatment regimens. Developing standardized approaches to using EHR data could help advance TB elimination in the U.S. by facilitating partnerships between primary care providers and public health.

## CRediT authorship contribution statement

**Laura A. Vonnahme:** Conceptualization, Methodology, Formal analysis, Writing – original draft, Writing – review & editing, Visualization, Supervision, Project administration. **Julia Raykin:** Methodology, Software, Formal analysis, Data curation, Writing – review & editing, Visualization. **Matthew Jones:** Software, Formal analysis, Data curation, Writing – review & editing. **Jee Oakley:** Conceptualization, Writing – review & editing, Project administration, Funding acquisition. **Jon Puro:** Conceptualization, Writing – review & editing, Supervision, Project administration, Funding acquisition. **Adam Langer:** Conceptualization, Writing – review & editing, Supervision. **Kaylynn Aiona:** Conceptualization, Methodology, Formal analysis, Writing – review & editing. **Robert Belknap:** Conceptualization, Methodology, Formal analysis, Writing – review & editing. **Tracy Ayers:** Conceptualization, Methodology, Formal analysis, Writing – original draft, Writing – review & editing, Visualization, Supervision. **Jonathan Todd:** Conceptualization, Methodology, Formal analysis, Writing – review & editing. **Kathryn Winglee:** Conceptualization, Methodology, Software, Formal analysis, Writing – original draft, Writing – review & editing, Visualization, Supervision.

## Appendix Supplementary materials

Image, application 1

## Footnotes

Supplementary material associated with this article can be found in the online version at doi:10.1016/j.focus.2023.100148.
