# Supplementary material for: Using Electronic Health Record Data to Measure the Latent Tuberculosis Infection Care Cascade in Safety-Net Primary Care Clinics
Source: AJPM Focus. 2023 Sep 28;2(4):100148. doi: 10.1016/j.focus.2023.100148 (PMC10630620; doi:10.1016/j.focus.2023.100148)
Supplement: Supplementary file 1 [file mmc1.docx]

**Appendix Figure 1. Defining diagnostic test results for patients in the latent tuberculosis infection care cascade using electronic health record data variables**

**
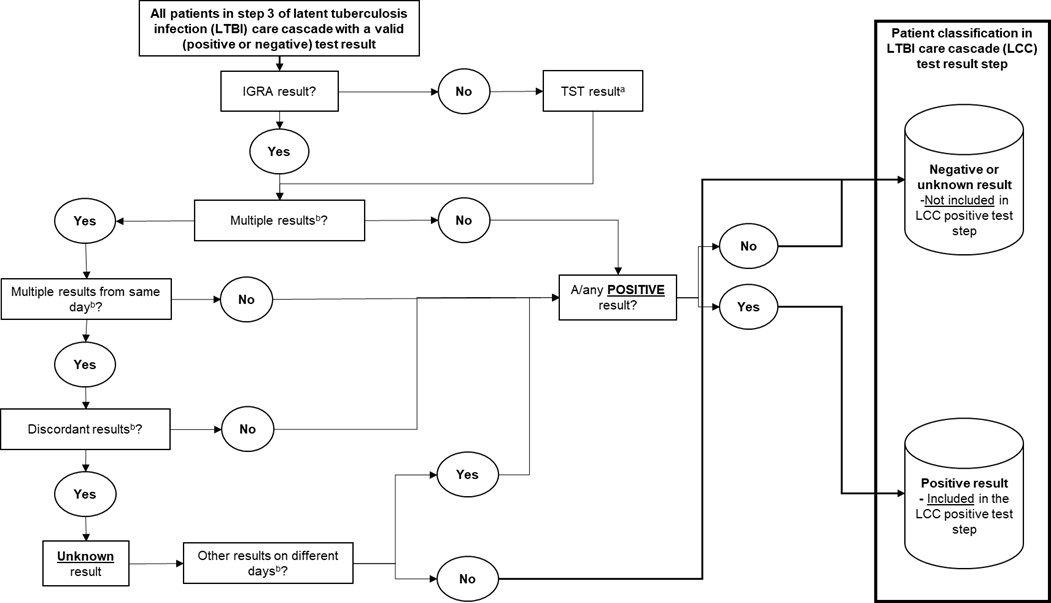
**

IGRA=interferon-gamma release assay; TST=tuberculin skin test

^a^Since this population has a known valid result (positive or negative) and individuals do not have a valid IGRA result at this point in logic, must have valid TST result

^b^Same test type (i.e., IGRA, TST)

**Appendix Methods**

**Algorithm for determining rifampin or rifabutin prescriptions as part of latent tuberculosis infection or disease treatment regimens**

Rifampin is used frequently to treat other infectious diseases^1^, thus a prescription for rifampin alone is not necessarily indicative that a patient is being treated for latent tuberculosis (TB) infection (LTBI) or TB disease. We created an algorithm for determining whether a rifampin or rifabutin prescription present in an EHR record was indicative of a LTBI or TB disease treatment regimen based on other drugs prescribed with rifampin or rifabutin, frequency and duration of rifampin or rifabutin prescriptions, and presence of other ICD-10 codes indicating conditions that are treated with rifampin or rifabutin (Appendix Table). This algorithm does not try to distinguish between rifampin or rifabutin prescribed for treatment of TB disease versus LTBI.

If rifampin or rifabutin is prescribed with isoniazid or pyrazinamide it is considered to be indicative of a TB or LTBI treatment regimen. If prescribed in the absence of isoniazid or pyrazinamide, with a dose frequency greater than once per day, the rifampin or rifabutin prescription is classified as not indicative of a TB or LTBI treatment regimen; if the frequency is once per day or unknown, but the duration is less than or equal to 30 days or over 365 days it is also considered to not be indicative of a TB or LTBI regimen. If rifampin or rifabutin is prescribed in the absence of isoniazid or pyrazinamide, with a frequency of once per day or unknown and a duration greater than 30 days but less than or equal to 365 days, then ICD-10 codes for other conditions treated with rifampin or rifabutin are considered. If there is an ICD-10 code for a condition typically treated with rifampin or rifabutin (Appendix Table 2) then the rifampin or rifabutin prescription is classified as not prescribed as part of a TB or LTBI regimen. However, if there is no evidence of an ICD-10 code for those conditions in the patient’s record, then it is assumed that the rifampin or rifabutin prescription is being prescribed as part of a TB or LTBI treatment regimen. This algorithm was created in close collaboration with TB clinicians and many resources related to LTBI and TB treatment regimens, drug dosing, and ICD coding were utilized^2–11^.

**References**

1. Prescribers’ Digital Reference (PDR): Rifampin - Drug Summary. Published 2022. https://www.pdr.net/drug-summary/Rifadin-rifampin-1036

2. Sterling TR, Njie G, Zenner D, et al. Guidelines for the Treatment of Latent Tuberculosis Infection: Recommendations from the National Tuberculosis Controllers Association and CDC, 2020. *Am J Transplant*. Published online 2020. doi:10.1111/ajt.15841

3. Centers for Disease Control and Prevention (CDC). CDC guidelines for treatment regimens for LTBI. https://www.cdc.gov/tb/topic/treatment/ltbi.htm

4. Nahid P, Dorman SE, Alipanah N, et al. Official American Thoracic Society/Centers for Disease Control and Prevention/Infectious Diseases Society of America Clinical Practice Guidelines: Treatment of Drug-Susceptible Tuberculosis. *Clin Infect Dis an Off Publ Infect Dis Soc Am*. 2016;63(7):e147-e195. doi:10.1093/cid/ciw376

5. Nahid P, Mase SR, Migliori GB, et al. Treatment of Drug-Resistant Tuberculosis. An Official ATS/CDC/ERS/IDSA Clinical Practice Guideline. *Am J Respir Crit Care Med*. 2019;200(10):e93-e142. doi:10.1164/rccm.201909-1874ST

6. Carr W, Kurbatova E, Starks A, Goswami N, Allen L, CA W. Interim Guidance: 4-Month Rifapentine-Moxifloxacin Regimen for the Treatment of Drug-Susceptible Pulmonary Tuberculosis — United States, 2022. *MMWR Morb Mortal Wkly Rep*. 2022;71:285-289. doi:http://dx.doi.org/10.15585/mmwr.mm7108a1external icon

7. Centers for Disease Control and Prevention (CDC). Treatment for TB Disease. Published 2022. https://www.cdc.gov/tb/topic/treatment/tbdisease.htm

8. Centers for Disease Control and Prevention (CDC). Provisional CDC Guidance for the Use of Pretomanid as part of a Regimen [Bedaquiline, Pretomanid, and Linezolid (BPaL)] to Treat Drug-Resistant Tuberculosis Disease. Published 2022. https://www.cdc.gov/tb/topic/drtb/bpal/default.htm

9. Curry International Tuberculosis Center and California Department of Health. *Drug-Resistant Tuberculosis: A Survival Guide for Clinicians, 3rd Edition/2022 Updates*.; 2022. https://www.currytbcenter.ucsf.edu/products/view/drug-resistant-tuberculosis-survival-guide-clinicians-3rd-edition

10. Prescribers’ Digital Reference (PDR). https://www.pdr.net/

11. Lexicomp. https://online.lexi.com/

**Appendix Table 1. Algorithm for determining rifampin or rifabutin prescriptions as part of latent TB infection or disease treatment regimens**

| **Prescribed with**  **isoniazid or pyrazinamide^a^** | **Frequency** | **Duration^b^** | **ICD-10 code for condition treated with rifampin^c^** | **Classification** |
| --- | --- | --- | --- | --- |
| Yes | NA | NA | NA | TB or LTBI |
| No | >1/day | NA | NA | not TB or LTBI |
| No | 1/day or unknown | duration <= 30 days | NA | not TB or LTBI |
| No | 1/day or unknown | duration > 365 days | NA | not TB or LTBI |
| No | 1/day or unknown | 365 days >= duration > 30 days or unknown | Yes | not TB or LTBI |
| No | 1/day or unknown | 365 days >= duration > 30 days or unknown | No | TB or LTBI |
| TB=Tuberculosis  LTBI=Latent TB infection  ^a^Prescribed the same day | | | | |
| ^b^Duration calculated using prescription quantity*(refills+1); if refills were missing assumed there were zero refills; if there was another prescription within 45 days then they were added together; if frequency is unknown, assumed frequency was 1 to calculate duration.  ^c^Anaplasmosis, bartonella infections, brucellosis, cholestatic pruritis, staph endocarditis, hidradenitis suppurativa, leprosy, meningococcal prophylaxis, pulmonary NTM severe and non-severe, staphylococcus infections, group A strep carriage (eTable 2). | | | | |

**Appendix Table 2. Non-TB or LTBI conditions treated with rifampin or rifabutin**

| **Indication** | **International Classification of Diseases (ICD)-10 codes** |
| --- | --- |
| Anaplasmosis | A79.82 |
| Bartonella infections | A44.x, A79.0 |
| Brucellosis | A23.x |
| Cholestatic pruritis | L29.8, L29.9 |
| Staph endocarditis | I33.0, T82.6x, T82.7x |
| Hidradenitis suppurativa | L73.2 |
| Leprosy | A30.x |
| Meningococcal prophylaxis | Z20.811 |
| Pulmonary nontuberculosis mycobacterium, non-severe | A31.x |
| Pulmonary nontuberculosis mycobacterium, severe | A31.x |
| Staphylococcus infections | B95.61, B95.62, B95.7, B95.8; M86.x; M00.0x, T84.5x, T84.6x, T84.7x; G00.3, G04.2, G06.x, G07, G08; R78.81; T85.7x; T81.4x; S91.x; L97.x; L89.x |
| Group A Strep carriage | Z22.338 |
